# Identification of novel modifiers of Aβ toxicity by transcriptomic analysis in the fruitfly

**DOI:** 10.1038/srep03512

**Published:** 2013-12-16

**Authors:** G. Favrin, D. M. Bean, E. Bilsland, H. Boyer, B. E. Fischer, S. Russell, D. C. Crowther, H. A. Baylis, S. G. Oliver, M. E. Giannakou

**Affiliations:** 1 Cambridge Systems Biology Centre, University of Cambridge, Cambridge, CB2 1GA, UK; 2 Department of Biochemistry, University of Cambridge, Cambridge, CB2 1GA, UK; 3 Department of Zoology, University of Cambridge, Cambridge, CB2 3EJ, UK; 4 Department of Genetics, University of Cambridge, Cambridge, CB2 3EH, UK; 5 These authors contributed equally to this work.

## Abstract

The strongest risk factor for developing Alzheimer's Disease (AD) is age. Here, we study the relationship between ageing and AD using a systems biology approach that employs a *Drosophila* (fruitfly) model of AD in which the flies overexpress the human Aβ_42_ peptide. We identified 712 genes that are differentially expressed between control and Aβ-expressing flies. We further divided these genes according to how they change over the animal's lifetime and discovered that the AD-related gene expression signature is age-independent. We have identified a number of differentially expressed pathways that are likely to play an important role in the disease, including oxidative stress and innate immunity. In particular, we uncovered two new modifiers of the Aβ phenotype, namely Sod3 and PGRP-SC1b.

The phenomenon of protein aggregation has been associated with a variety of human disorders that affect large sections of the population worldwide^1^,^2^,^3^. These disorders, which include Alzheimer's, Parkinson's, type II diabetes, and the spongiform encephalopathies, are rapidly becoming one of the most important groups of pathologies worldwide in terms of both incidence and social costs.

Alzheimers disease (AD) is the leading cause of dementia in the human population. At least for familial AD, mutations that result in the generation of aggregation-prone isoforms of the amyloid β peptide are sufficient to cause amyloid plaques in the brain and the clinical features of the disease. Mature amyloid plaques are always seen in AD; however it is thought that precursor conformers, termed Aβ oligomers, are of primary importance in the pathology. It is becoming evident that, while the neuronal injury in AD is initiated by the accumulation of neurotoxic aggregates of Aβ peptide, these then give rise to a complex network of downstream events, (including aggregation of the tau protein) that culminate in neurodegeneration^3^. Neither the complete list of pathways involved in disease progression nor the causal chain of events that unites them is clear. It is therefore becoming increasingly apparent that a paradigm shift is required in order to describe and rationalise this complexity. It may simply not be possible to understand sporadic AD as the result of perturbations to a single pathway, where a single cause leads to the effect. Rather, the disorder should be studied as a system; that is, a change in the homeostatic equilibrium of many pathways^4^.

Ageing is a physiological process rather than just a chronological one. It is accompanied by changes in the steady-state mRNA levels of a number of genes and in the levels of many proteins that are involved in a variety of physiological processes^5^. A component of ageing is the collapse of cellular protein homeostasis and this process is thought to underpin the increasing incidence of protein aggregation diseases in the elderly^4^,^6^. Indeed, age-related changes in gene transcription lead to decreased quality control functions with age^7^. Late onset Alzheimer's disease accounts for the overwhelming majority^8^ of disease cases, making age the strongest risk factor for developing the disease.

It follows that, in order to elucidate the underlying causes that trigger AD and affect its development, it is necessary to investigate the changes in AD as a function of age. Here, we have used a *Drosophila* model of AD^9^ to study how various cellular pathways (as measured by transcription profiling) change with age and AD. In this model, the secreted human Aβ_42_ peptide is expressed specifically in the central nervous system of *Drosophila melanogaster*. The model recapitulates many of the pathologies observed in human AD, including Aβ accumulation, and premature death^9^,^10^,^12^. In the present study, we have used two versions of this model. For the transcriptomics study, we expressed the wild type Aβ_42_ coding sequence whereas, for gene-specific RNAi knock-down or over-expression experiments, we extended our study by validating the original observations in flies expressing the familial AD-linked Arctic (E22G) variant of Aβ_42_. We investigated the changes in transcriptome profiles over time for both control flies and those expressing Aβ_42_. The use of such an early onset model allowed us to distinguish between changes in gene expression due to AD and those due to ageing.

## Results

### Transcriptome analysis of AD and control flies over time

**Figure 1:**
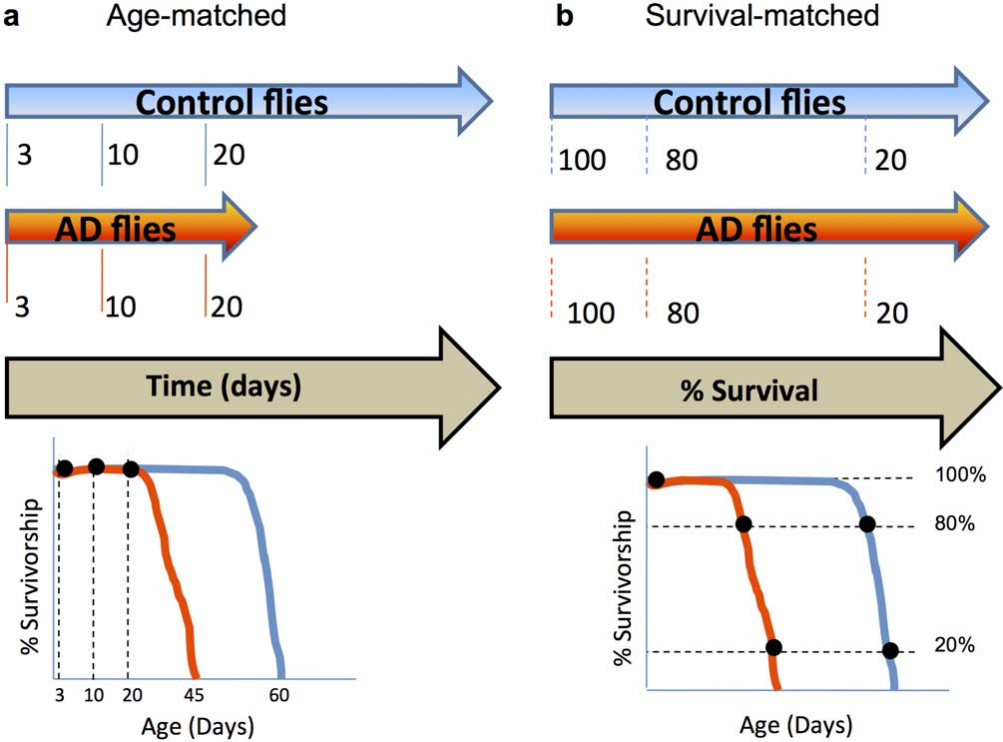
Schematic representation of the experimental design. (a) RNA samples are extracted from both control and AD flies at the same time in days. (b) RNA samples are extracted from both AD and control flies at the same % survival, which is a different time in days for each after 100% survival.

To investigate the differences between the processes of ageing and AD we used microarrays to measure changes in gene expression over time in control and Aβ_42_-expressing flies (hereafter referred to as Aβ flies). The Aβ flies used in these experiments carried 2 copies of a transgene expressing human *Aβ_42_* (elavGAL4 > *UAS-Aβ_42_*), the *2* × Aβ_42_ model. Aβ flies have a much shorter lifespan with a median survival (50% flies still alive) of 23 *vs.* 63 days in control flies (see Supplementary Fig. S1, for the climbing, survival, and molecular phenotypes of Aβ flies used in our experiments). Therefore, in order to clearly distinguish Aβ and age-related changes in gene expression, we compared Aβ and control flies using a two-pronged strategy. In the first experiment, we age-matched flies according to their chronological age and extracted RNA samples from fly heads at days 3, 10 and 20 for both Aβ flies and the control cohort (Fig. 1). At these time points the survival of the flies is approximately 100% and so we can match samples from flies according to their chronological age.

Beyond day 20, mortality in Aβ flies begins to increase and it is conceivable that increasing mortality itself could be associated with changes in gene expression. To compare gene expression changes associated with the increase in mortality in Aβ flies with those associated with normal ageing, we continued to extract RNA samples from Aβ and control flies but at different times such that the % survival of each group was the same (80% and 20% survival, this corresponded to days 21, and 25 in Aβ flies and days 56, and 68 for control flies). We used this data for a separate analysis of the gene expression changes over time according to survival. All RNA samples were extracted from one cohort of flies (see Methods) to reduce the effect of biological variability, but the age-matched and survival-matched samples were analysed separately and we refer to them as separate experiments. The day 3 (100% survival) sample is common to both analyses.

**Figure 2:**
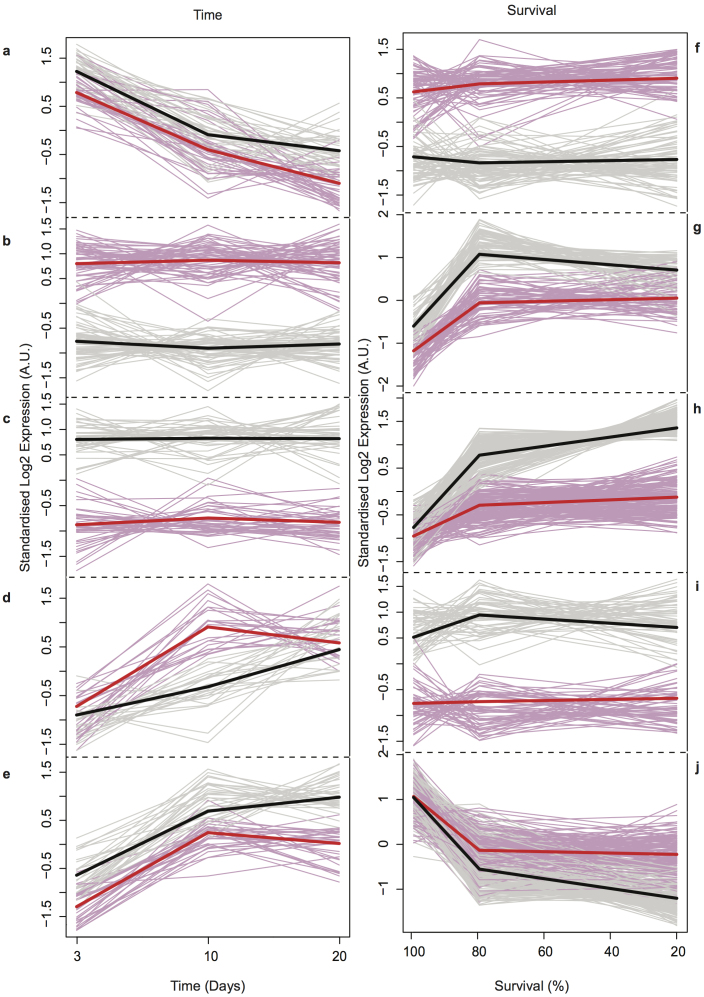
Clustering of changes in gene expression in Control and Aβ flies. (a–j). Clustering of gene expression changes in Control (black) and AD (red) using Mfuzz. Bold lines represent cluster centroids, thin lines are average expression profiles for all cluster members plotted against equivalent time points (a–e) based on day (days 3, 10 and 20 for both Aβ and control flies, all approximately 100% survival) and (f–j) based on % survival of the flies (days 3, 21 and 25 (Aβ flies) or days 3, 56 and 68 (control flies), corresponding to 100%, 80% and 20% survival in each case).

Analysis of the gene expression profiles from the two experiments (see Methods for details) identified 233 (day-matched) and 636 (survival-matched) differentially expressed genes, with a total of 712 genes combined (and an overlap of 157 genes, see Supplementary Table S1 online). We clustered average expression levels of the significantly differentially expressed genes using fuzzy c-means clustering (R mfuzz package)^12^,^13^ for each of the two experiments (Fig. 2: “a–e” day-matched and “f–j” survival-matched).

In each experiment, we identify two categories of clusters. The first category of clusters (for both experiments), represents genes that are differentially up- or down- regulated in Aβ flies compared to controls, but do not change with time (Clusters “b”, “c”, “f” and “i”; 69, 47, 95 and 85 genes respectively – note that as fuzzy clustering is used, the number of genes assigned to each cluster does not total the number of significant genes). These genes presumably represent a direct response of the flies to the Aβ aggregation insult. By contrast the second category of expression profile clusters, for both experiments, represents genes with expression profiles that change over time (clusters “a”, “d”, “e”, “g”, “h” and “j” - 46, 35, 39, 121, 169, and 171 genes respectively). Based on these clusters, it appears that very few genes change expression over time in Aβ flies compared to controls, and that changes over time are more pronounced in control flies.

We therefore analysed the expression profiles of all genes present on the array for Aβ and control flies separately (see Methods) to identify all genes that change expression significantly over time in each group. For Aβ flies we identified 144 genes, whereas for control flies we identified 612 (with an overlap of 90 genes, Supplementary Table 1 online). For both Aβ and control flies, the genes changing over time were involved in similar pathways based on enriched Gene Ontology (GO) terms, in particular immune response and metabolic processes (data not shown). Therefore we conclude that the dysregulation is gene specific rather than pathway specific. For each of these genes, we tested the correlation between gene expression level and percent survival. Of the 144 genes whose expression level changes significantly over time in Aβ flies, there were only four (IM23, CG14933, CG7830, CG8036) whose expression level correlated with survival (Pearson's Product Moment Correlation Coefficient (*ρ*), 0.8 ≤ *ρ* ≤ −0.8). There is little information available on the function of these four genes, so we are unable to explain why the expression of these genes in particular correlates with the decrease in survival. Based on the fact that only 144 genes change expression over time in Aβ flies, we conclude that the transcriptional response to Aβ expression in our fly model is mostly not age dependent.

By contrast, 195 out of 612 genes changing expression correlated with the decreased survival in control flies using the same threshold. Of the four genes whose expression correlated with survival in Aβ flies, two were also correlated in controls (CG14933 and IM23) and the expression of both genes increased over time for both Aβ and control cohorts. We therefore conclude that there is no common mortality signature (i.e. genes that change in expression over time in both AD and control cohorts and whose expression levels correlated with survival in both), and that the gene expression changes occurring with normal ageing are distinct from those associated with Aβ expression in *Drosophila*. At the transcriptional level, the Aβ expression-associated signature is a constant change in the relative level of transcription of a certain set of genes over all time points measured, rather than the signature of ageing that manifests as a change in the level of transcription over time.

The 612 genes whose expression changes significantly over time in our control flies constitute a transcriptional signature of normal ageing in control flies. We compared this list of genes to a previous ageing study in *Drosophila*^14^. 44% (282/612) of genes changing expression with age in our control flies were also identified as ageing-related genes by Landis *et al*^14^, significantly more than expected by chance (hypergeometric test for enrichment, p < 1 × 10e-19). Furthermore, 63% of our 195 ageing signature genes for which expression level correlated with decreased survival with age were identified as ageing genes in the same study^14^ (hypergeometric test for enrichment, p < 1 × 10e-22). An analysis of the correlated genes for the control cohort using the Flymine database^15^ revealed that the pathways in which these genes are involved are significantly enriched in a number of pathways and GO annotations related to xenobiotic metabolism, glutathione metabolism and immune response pathways^16^,^17^. This is consistent with current theories of ageing^19^,^20^,^18^. The transcriptional changes associated with ageing in our control flies are therefore consistent with expected changes with age in *Drosophila*, both at the single gene and pathway level. The fact that our analysis produces expected results for control flies lends weight to the conclusions drawn from our analysis of the Aβ flies.

We performed Principal Components Analysis (PCA) of the expression data (Supplementary Fig. S2) and found that, while across the first three sample points Aβ and control groups cluster together, in the final two (where samples did not have the same age in days) they do not, confirming that the downstream effects of Aβ expression in this model are not similar to normal ageing, at least at the level of transcription. Supplementary Table S1 online lists all the differentially expressed genes, what method determined their significance, the cluster to which they belong for both experiments, their Pearson correlation with survival and their normalised expression level (see Methods) for each data point and for each of the replicates.

### Oxidative stress-related changes in gene expression with AD

**Figure 3:**
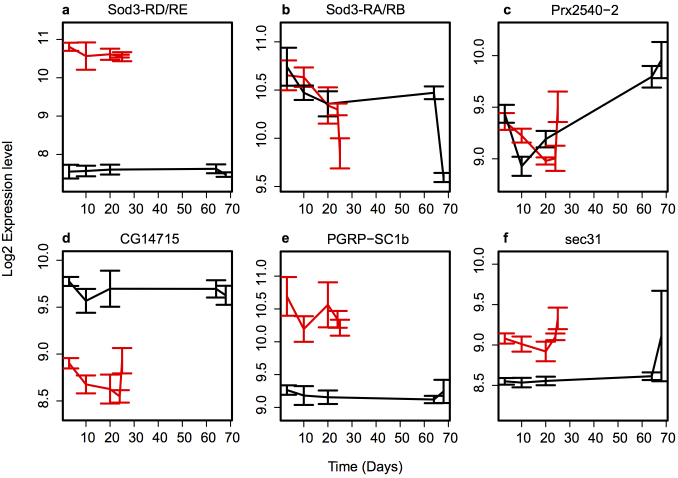
Expression profiles of individual genes in Control and Aβ flies. Gene expression profiles of RNA from heads from Control (black) and Aβ (red) female mated flies at 6 time points during adulthood. Time points correspond to days 3, 10 and 20 (100% survival) in both control and Aβ flies and at 56 (21), and 68 (25) days in Control (Aβ) flies that correspond to 80% and 20% survival, respectively. Average gene expression levels of four biological replicates (except time point 3 and 5 which are the average of 3 biological replicates, see Methods) ± s.e.m. plotted. (a) *Sod3-RD and Sod3-RE* (b) *Sod3-RA and Sod3-RB* (c) *Prx2540-2* (d) *CG14715* (e) *PGRP-SC1b* (f) *sec31*.

The transcript most highly up-regulated (~8-fold; Fig. 3a) in Aβ flies in our experiments was *Sod3* (CG9027) which encodes an extracellular Cu/Zn superoxide dismutase^21^. In *Drosophila* there are 4 *Sod3* transcripts (and 3 protein products), and two of these, *Sod3-RD* and *Sod3-RE* are specifically up-regulated in the Aβ flies (see Fig. 3a for *Sod3-RD/RE* and Fig. 3b for *Sod3-RA/RB* profiles). Superoxide dismutase enzymes have recently been linked to inflammation, with *Sod3* proposed to contribute to this process both by scavenging free radicals and also, more directly, by affecting immune responses and signal initiation^22^. Interestingly, in 2009, before *Sod3* had been discovered in flies^21^, Rival et al^10^ showed that overexpression of Sod1 was associated with a decrease in lifespan in Aβ flies.

*Cyp6a20*, encoding a cytochrome P450 enzyme, was another gene with significantly altered expression in Aβ flies compared to control flies. We found that *Cyp6a20* expression was significantly reduced in Aβ flies in an age- independent manner (see Supplementary Table S1 for expression data). *Cyp6a20* was also identified in a genetic screen as a modifier of the survival phenotype in Aβ flies^10^ and cytochrome P450s have previously been identified as being a group of enzymes that are up-regulated with age^24^,^23^. On the other hand, genes such as *Prx2540-2* (Fig. 3c), encoding peroxidases involved in the clearance of hydrogen peroxide^25^, appear to change with time in control flies. This change was not observed in Aβ flies. Increased expression with age in control, but not Aβ, flies was also observed for genes involved in glutathione metabolism, for example several of the glutathione-S-transferases increased expression over time only in control flies (Supplementary Table S1). Interestingly, one member of this family, GstE9, did increase expression over time in Aβ flies. We again conclude that the same processes are important in Aβ toxicity and ageing, but with different specific genes affected by each process.

### Immune response-related changes in gene expression with AD

Two important processes that showed significantly altered gene expression profiles in our AD model are the innate immunity and defence response pathways. Many genes in these pathways normally become up-regulated during ageing^23^,^26^. In control flies, increased expression with age was observed for genes involved in the antibacterial humoral response^14^, such as *Dpt*, *CecA1*, *CecA2* and *Drs.* Similar changes were not observed in Aβ flies. For example, immune response genes, such as that encoding the proteoglycan recognition protein PGRP-SC1b were significantly altered in our experiment (Fig. 3e), with increased transcript levels in Aβ flies *vs.* controls at all time points. PGRP-SC1b is a catalytic PGRP that is likely to be involved in down-regulation of the Imd innate immunity pathway in response to injury^27^.

### Cellular transport and chaperone related changes in gene expression with AD

Intracellular transport, specifically endocytic processing, has been implicated in AD using GWA studies for sporadic AD^28^. In our study, *sec31*, a gene coding for an essential component of the COPII coat for ER to Golgi transport^29^, was increased in expression in Aβ flies compared to controls (Fig. 3f). *CG14715*, another gene implicated in intracellular transport and protein folding, encodes the *Drosophila* ortholog of *FKBP2/FKBP13*, a prolyl-isomerase thought to function as an ER chaperone^31^,^30^. The expression of *CG14715* was down-regulated in an age-independent manner in the Aβ flies (Fig. 3d).

### Identifying modifiers of the Aβ phenotype using gene-specific RNAi

Our microarray analysis identified significant expression changes (between control and Aβ flies) in 712 genes. These genes are potential modifiers of the Aβ phenotype. In order to determine whether the observed expression changes are relevant to the onset or development of the disease or whether they are simply correlative, we manipulated the levels of four of these genes in the *Drosophila* AD model (Data summarised in Table 1). We chose two genes that are highly up-regulated in an age-independent manner in Aβ flies: *Sod3*, which was identified as the most differentially expressed gene between the Aβ and control flies (~8 fold increased levels in Aβ flies, Fig. 3a) and *PGRP-SC1b* (~2 fold increased levels in Aβ flies, Fig. 3e). These genes are involved in oxidative stress and innate immunity, respectively. We also investigated *sec31*, encoding a protein involved in intracellular transport (~1.5 fold increased levels in Aβ flies, Fig. 3f) and *CG14715*, coding for a putative ER chaperone (~1.7 fold decreased levels in Aβ flies, Fig. 3d).

In these experiments, we used a modified version of the fly AD model (*elavGAL4* *>* *UAS- Aβ_42_arc*) in which flies expressed a single copy of the Aβ peptide containing the familial E22G (arctic) mutation that increases the aggregation propensity of the Aβ_42_ peptide^32^. It has been shown previously^11^,^32^,^33^ that these models are equivalent and that their effect on the flies' lifespan is proportional to the aggregation propensity of the Aβ variant. Luheshi et al. (2010) investigated the effect of mutations (including the arctic mutation) in the sequence of Aβ_42_ and showed that the aggregation propensity and *in vivo* toxicity as quantified by locomotor and survival assays were correlated. Finally, as shown below, the two models show similar changes in expression of specific genes. Although, we cannot exclude the possibility that the brain pathology due to expression of the alternative Aβ transgenes is different, this evidence suggests that they have similar effects.

**Figure 4:**
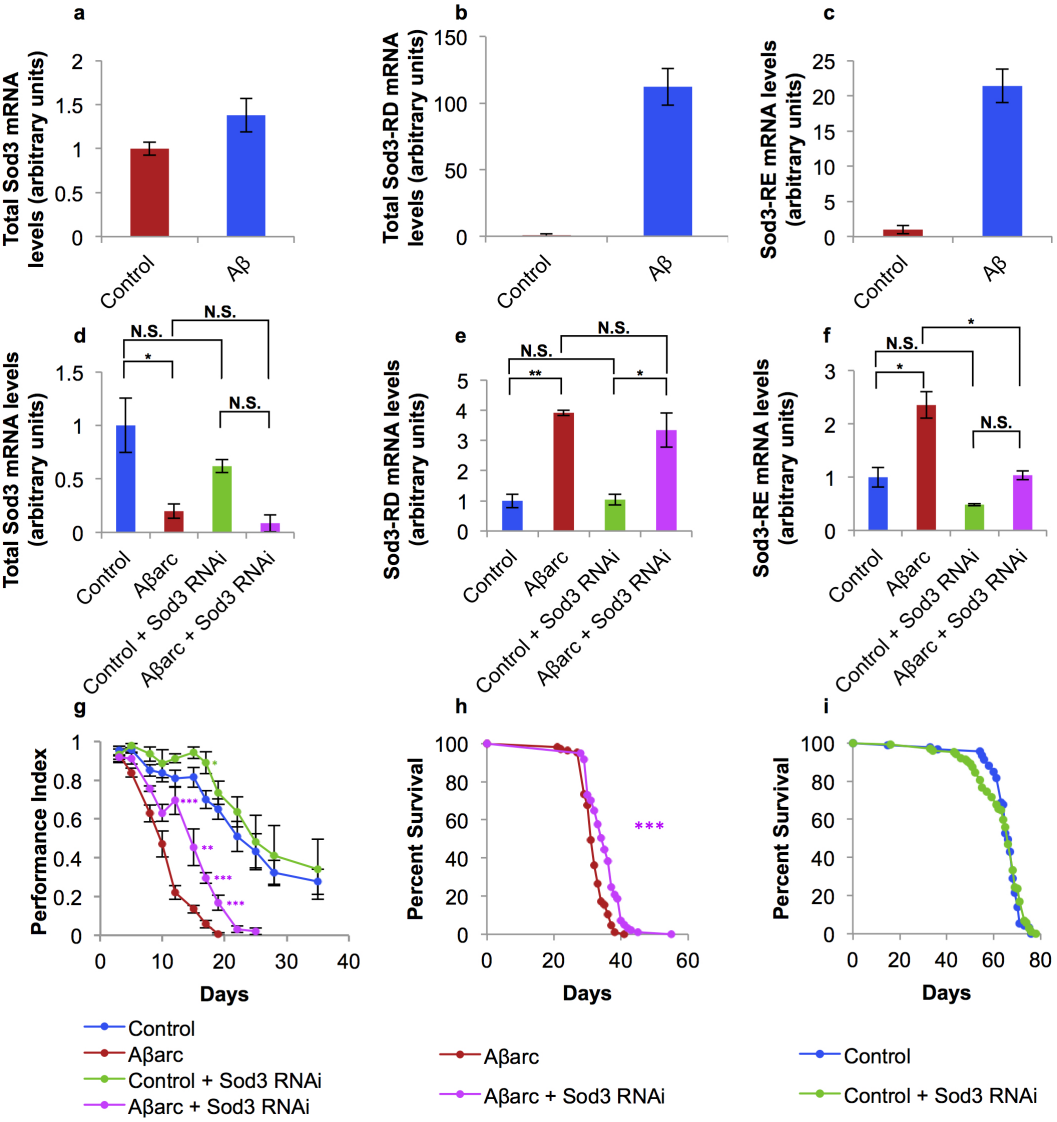
Sod3 levels in Aβ flies and effects of *Sod3* RNAi on *Sod3* expression, locomotion and survival. (a–c). Levels of *Sod3* transcripts in head RNA from Aβ (*elavGAL4* *>* *UAS-Aβ42* × 2) and control (*elavGAL4*/+) female mated flies at day 20 measured by qRT-PCR and plotted relative to *Act5C* mRNA levels, in arbitrary units, represented relative to levels in control (set to 1). (a) Total *Sod3* mRNA levels (primers against A, B, D, E common transcript). (b) *Sod3-RD* mRNA levels. (c) *Sod3-RE* mRNA levels. (d–f), Levels of Sod3 transcripts in head RNA from Control (*elavGAL4*/+), Aβarc (*elavGAL4* *>* *UAS-Aβ42arc*), Control + Sod3 RNAi and Aβarc + Sod3 RNAi female mated flies at day 7 measured by qRT-PCR and plotted relative to *Act5C* mRNA levels, in arbitrary units, represented relative to mRNA levels in Control (set to 1). qRT-PCR analysis: n = 3 replicates, mean ± s.e.m. plotted. mRNA levels between groups compared using two-tailed Student's *t*-test (*f*-test for equal variance). (d) Total *Sod3* mRNA levels. (e) *Sod3-RD* mRNA levels. (f) *Sod3-RE* mRNA levels. (g) Climbing performance of Control (*elavGAL4*/+), Aβarc (*elavGAL4* *>* *UAS-Aβ42arc*), Control + Sod3 RNAi and Aβarc + Sod3 RNAi mated females at different time points at 24°C. n = 3 (3 replicates, 10 flies/replicate). Performance indices (see Methods) between Control and Control + Sod3 RNAi and Aβarc and Aβarc + Sod3 RNAi were compared at each time-point using two-tailed Student's *t*-test (*f*-test for equal variance). (h) Survival curves of Aβarc (n = 105, median = 31, P < 0.0001 *vs.* Control) and Aβarc + Sod3 RNAi (n = 97, median survival = 35, P < 0.0001 *vs.* Aβarc) mated females at 25°C. (i) Survival curves of Control (n = 93; median survival = 66) and Control + Sod3 RNAi (n = 102; m = 66, P = 0.5280 *vs.* Control) mated females at 25°C. Comparison of survival curves was carried out using the Log-rank test. P values: *, P < 0.05; **, P < 0.01; ***, P < 0.0001.

We confirmed the up-regulation of *Sod3* using qRT-PCR. *Sod3* has 4 alternative transcripts. Increases in *Sod3* mRNA levels in the microarray experiment (neuronal expression of two copies of Aβ_42_) were specific to two transcripts, *Sod3-RD* and *Sod3-RE*. qRT-PCR quantification of total *Sod3* levels suggested a 50% increase in *Sod3* transcription in the heads of Aβ flies at day 20 (Fig. 4a). However, transcript-specific primers showed a much larger increase in *Sod3-RD* (~100-fold) and *Sod3-RE* levels (~20-fold) confirming the array data (Fig. 4b, 4c). In Aβarc flies (expressing neuronal *Aβ_42_arc* peptide), total *Sod3* mRNA levels were actually decreased (Fig. 4d). However as in the 2 × Aβ_42_ flies, both *Sod3-RD* and *Sod3-RE* mRNA levels were significantly increased in heads from Aβarc flies (Fig. 4e, 4f). Thus, both Aβ and Aβarc flies show a specific increase in the levels of *Sod3-RD* and Sod3*–RE* transcripts.

As *Sod3* transcript levels are also increased in Aβarc flies, we tested whether reducing *Sod3* levels using RNAi could ameliorate the Aβ phenotype. Ubiquitous RNAi against *Sod3* resulted in effective knock-down of total *Sod3* expression levels in head mRNA (down to 10% of control) and substantial knock-down of *Sod3-RD* and *Sod3-RE* expression levels (down to 30–40% of control: Supplementary Fig. S3), confirming that indeed the Sod3-RNAi line is substantially knocking down *Sod3* transcript levels.

Targeting *Sod3* RNAi specifically to the nervous system resulted in a consistent (though not statistically significant) decrease in total *Sod3* RNA levels in head RNA from both *Sod3* RNAi control and Aβarc flies (*vs.* non-RNAi controls, Fig. 4d–f). Since *Sod3* levels were measured from whole head RNA extracts, which also include other cell types apart from neurons, our measurement of total head *Sod3* mRNA is likely to be an underestimate of the actual knock-down in the nervous system.

*Sod3* RNAi improved climbing (Fig. 4g and Supplementary Fig. S3) and survival in AD flies; median survival for Aβarc *Sod3*-RNAi flies was 35 days *vs.* 31 days for control Aβarc flies (Log-rank test, P < 0.0001: Fig. 4h and Supplementary Fig. S3), and this appeared to correlate with significantly decreased *Sod3-RE* mRNA levels (Fig. 4f). Ubiquitous knock-down of *Sod3* has previously been reported to be detrimental for *Drosophila* lifespan^21^. However, we did not observe any significant effect of nervous system-specific *Sod3*-RNAi in control (*i.e.* no Aβarc) flies (Fig. 4g and 4i and Supplementary Fig. S3). Thus, ablating *Sod3* up-regulation in Aβarc flies ameliorates the Aβ phenotype.

**Figure 5:**
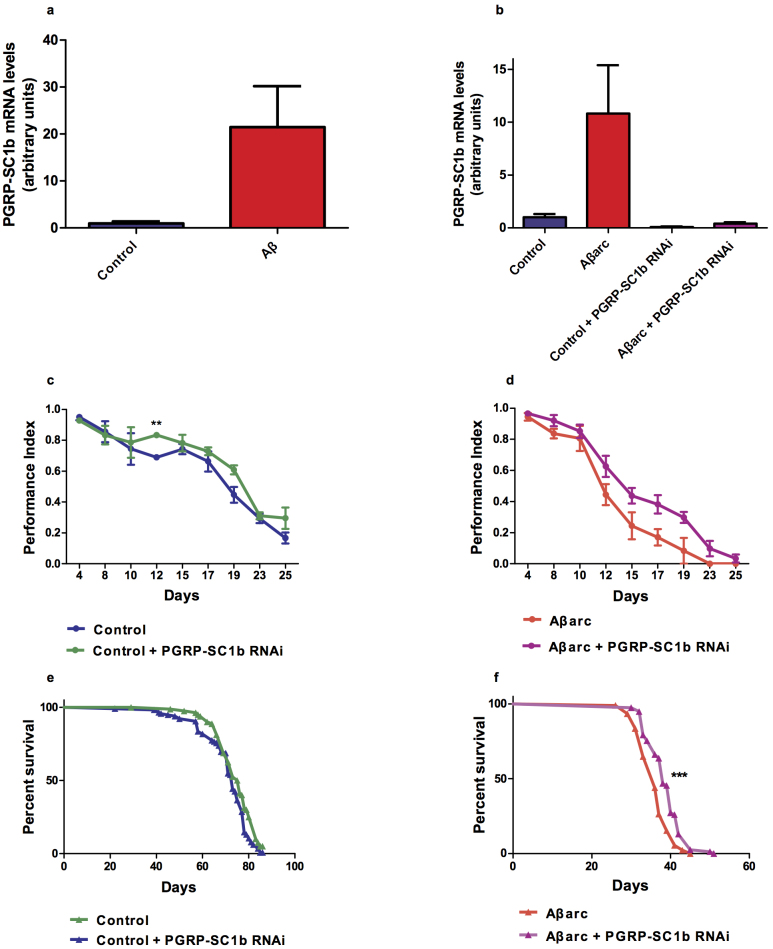
Effects of *PGRP-SC1b* RNAi on *PGRP-SC1b* expression, locomotion and survival. (a) PGRP-SC1b mRNA levels in head RNA from Aβ (*elavGAL4 > UAS-Aβ42* × 2) and control (*elavGAL4*/+) mated female flies at day 20 measured by qRT-PCR and plotted relative to *Act5C* mRNA levels, in arbitrary units, represented relative to mRNA levels in elavGAL4/+ flies (set to 1). n = 3 replicates, mean ± s.e.m. plotted. mRNA levels between groups compared using two-tailed Student's *t*-test (*f*-test for equal variance). (b) Levels of *PGRP-SC1b* mRNA in head RNA from Control *(elavGAL4/+)*, Aβarc *(elavGAL4>UAS-Ab42arc)*, Control + PGRP-Sc1b RNAi, and Aβarc + PGRP-SC1b RNAi from flies at day 7 measured by qRT-PCR. n = 3 replicates, mean ± s.e.m. plotted. mRNA levels between groups compared using two-tailed Student's *t*- test (*f*-test for equal variance). (c) Climbing performance of Control, and Control + PGRP-Sc1b RNAi, at different time points at 24°C. Performance indices were compared at each time-point using two-tailed Student's *t*-test (*f*-test for equal variance). (d) Climbing performance of Aβarc and Aβarc + PGRP-SC1b RNAi at different time points at 24°C. Performance indices were compared at each time-point using two-tailed Student's *t*-test (*f*-test for equal variance). (e) Survival curves of Control (n = 119, median = 73) and Control + PGRP-SC1b RNAi (n = 77, m = 74.5, P = 0.1825 *vs.* Control) female flies at 25°C. (f) Survival curves of Aβarc (n = 91, median = 36, P < 0.0001 *vs.* Control) and Aβarc + PGRP-SC1b RNAi (n = 77, m = 38, P < 0.0001 *vs.* Aβarc) female flies at 25°C. Comparison of survival curves was carried out using the Log-rank test. P values: *, P < 0.05; **, P < 0.01; ***, P < 0.0001.

We next took a similar approach to investigating *PGRP-SC1b*. In the microarray study, *PGRP-SC1b* RNA levels were increased ~2-fold in Aβ flies (Fig. 3e); moreover, qRT-PCR, showed a similar increase (Fig. 5a). In Aβarc flies (expressing neuronal *Aβ_42_arc* peptide), *PGRP-SC1b* RNA levels were increased ~10-fold in head RNA (compared to control flies, Fig. 5b). Ubiquitous RNAi against *PGRP-SC1b* resulted in effective knock-down of *PGRP-SC1b* expression levels (down to 30% of control: Supplementary Fig. S5). Targeting RNAi against *PGRP-SC1b* to the nervous system resulted in improved climbing (Fig. 5d) and survival (median survival for Aβarc *PGRP-SC1b* RNAi flies was 38 days *vs.* 36 days for Aβarc, Log-rank test, P < 0.0001, Fig. 5f) flies compared to their (non-RNAi) controls. However, we did not observe any significant effect of nervous system-specific *PGRP-SC1b*-RNAi in control (*i.e.* no Aβarc) flies (Fig. 5c, 5e and Supplementary Fig. S4).

RNAi against *CG14715* (which encodes a prolyl-isomerase thought to function as an ER chaperone^31^,^30^) in the fly nervous system of Aβarc flies resulted in a significant increase in survival (median survival for two Aβarc CG14715 RNAi lines was 34 days *vs.* 31 days for Aβarc, Log-rank test, P < 0.0001; Supplementary Fig. S5) but no improvement in climbing ability (compared to non-RNAi controls; Supplementary Fig. S6). By contrast, overexpression of *CG14715*, in Aβarc flies, resulted in a significant increase in climbing ability in early adulthood but had no effect on survival (Supplementary Fig. S5). In control (non-Aβarc) flies, RNAi knock-down or overexpression of *CG14715* resulted in either no effect or a slight deficit in climbing ability; neither treatment affected survival (Supplementary Fig. S5). Thus manipulating *CG14715* expression can modify the Aβ phenotype, but the effect is complex.

Overexpression or RNAi knock-down of *sec31* in the fly nervous system resulted in decreased climbing ability and longevity in control (non-Aβarc) and either no, or a negative, effect in Aβarc flies. (Supplementary Fig. S6). The sec31 protein is an essential component of the COPII tracking complex^29^ so it likely that sec31 levels are critical for normal cellular function. Thus it is unclear whether sec31 has any specific modifying effect on the Aβ phenotype.

These results suggest that modifying expression of *Sod3*, *PGRP-SC1b and CG14715* in Aβ flies can suppress the locomotor and survival defects associated with toxic Aβ_42_ expression.

## Discussion

In this study, we have used time-course transcriptomic analysis to identify 712 *D. melanogaster* genes that are differentially expressed between Aβ-expressing and control flies. Our results suggest that Aβ-expressing flies are more similar to young than to old control flies. Since Aβ and control flies remained transcriptionally distinct as their mortality increased, we therefore conclude that the expression of Aβ in this model does not equate to an increased rate of ageing. Cluster analysis revealed that differentially expressed genes can be separated into those that change expression over time and those whose expression is constant. These results are consistent with the very aggressive nature of the model in which very high levels of Aβ are expressed at all times. We suggest that any change at the molecular level that correlates with the phenotype in the Aβ flies lies downstream of the transcriptome and that, by day 3, the Aβ flies are already in an essentially “terminal” transcriptional state. In this state, certain pathways such as the oxidative stress response pathway appear to be dysregulated and drive degeneration, which is expressed as both decreased locomotor activity and increased mortality.

We have further investigated some of the genes that were consistently over expressed in Aβ flies and we identified two modifiers of Aβ toxicity: *Sod3* and *PGRP-SC1b*. In particular we found that increased levels of two *Sod3* transcripts in Aβ flies were not accompanied by a compensatory increase in expression of either catalase or peroxidases. An imbalance in the relative levels of these three types of enzymes may result in an increased level of toxic H_2_O_2_ in Aβ flies, contributing to disease pathology. The expression of an RNAi for *Sod3* resulted in a reduction in mRNA levels for at least one *Sod3* transcript, and was accompanied by improved locomotor ability and survival in Aβ, but not control, flies. This suggests that decreasing Sod3 enzyme levels in our Aβ flies alleviated a toxic H_2_O_2_ overload. Rival *et al*^10^, found similar results regarding the toxicity of H_2_O_2_. In particular, they observed increased survival of Aβ flies when a dominant negative mutant *Sod1* was expressed and reduced survival when wild-type *Sod1* was expressed. By contrast, they found that the median survival was increased when *Cat* (encoding catalase) was overexpressed, suggesting that overproduction of H_2_O_2_ by the Sod1 enzyme can overwhelm catalase resulting in toxicity and a decrease in lifespan of the Aβ flies. Moreover, a previous study^34^ in *C. elegans* showed that loss of the *sod-4* gene, (the *C. elegans* ortholog of *Sod3*) had no effect on lifespan in wild-type worms, but increased the survival of *daf-2* (insulin receptor) mutants. Doonan and colleagues^34^ suggested that the SOD-4 enzyme may be generating H_2_O_2_, which acts as a signalling molecule and activates IIS (insulin/IGF-like signalling) by inactivating redox-sensitive phosphatases^35^; consequently, its loss in IIS mutants would enhance their long-lived phenotype. Increased H_2_O_2_ in Aβ flies could also act as a signal. Thus, by reducing Sod3 enzyme levels, we would decrease both the toxic overload and affect the signalling role of H_2_O_2_ to enhance lifespan. There have also been a number of reports on the activation of autophagy by H_2_O_2_ through the PI3K/Beclin1 and the PI3K/Akt/mTOR pathways^36^. In the case of PGRP-SC1b, it is not clear why RNAi results in an increase in lifespan and improved locomotor abilities in AD flies. We speculate that it could be due to a dysregulation of the pathway, similar to what we observed for *Sod3*.

The comparative analysis of gene expression between AD and ageing revealed changes at the single gene level, rather than the pathway level. Nevertheless, if the individual genes that are regulated very differently in Aβ flies *vs.* wild-type controls are considered, a number of important inferences may be made concerning AD. One particular example is the oxidative stress pathway, which is up-regulated with age in control flies (in our experiment and Landis et al)^14^. In the wild-type fly, manipulating cellular antioxidant defences (using transgenes) is not necessarily beneficial or detrimental to the health of the organism^18^. In other words, physiological levels of ROS can be dealt with by the insect's powerful enzymatic and non-enzymatic detoxification routes. However, mitochondrial dysfunction is observed in AD and this may be exacerbated with age^37^; therefore it is possible that ROS generated as a consequence of this mitochondrial dysfunction overwhelms cellular detoxification pathways.

It is becoming clear that the cascade of events that originates from the aggregation of Aβ and tau involves major stress response pathways, and all these stress pathways appear to be inter-related. However, their co-regulation and inter-relationships have been poorly characterised to date. In this study, we observed the dysregulation of a number of genes that belong to pathways that appear to be related, even though we were not able to assess their precise regulatory relationship. In all, this study has allowed us to investigate the processes that change in Aβ flies and dissect these from the processes that change with normal ageing. We observed a large number of dysregulated processes in AD flies. In particular, we highlight a number of genes involved in redox stress, innate immune response and pathogen defense response and intracellular transport. We have shown that either knocking-down or over-expressing some of these genes increased lifespan and improved locomotor (climbing) ability in Aβ flies compared to control flies. This suggests that the processes of oxidative stress and the immune response are likely to play an important role in the disease. The insights into time/age-dependent gene expression levels in AD that have been gained using an insect model may prove valuable in the design of strategies to combat this economically and socially important disease.

## Methods

### Fly stocks and maintenance

The *UAS-Aβ_42_-51D* and *UAS-Aβ_42_arc-51D* flies were generated using the PhiC31 method as previously described^38^. *ElavGAL4^C155^* and *tubGAL4* were used for neuronal-specific expression and ubiquitous expression of transgenes respectively. For microarray experiments, *w^1118^, elavGAL4* *> UAS-Aβ_42_-51D* (×2 copies) flies and controls *w^1118^, elavGAL4/+; 51D* (×2 copies, empty insertion site) were used. For RNAi experiments, we replaced *UAS-Aβ_42_-51D* (×2 copies) flies with flies carrying a single *UAS-Aβ_42_arc-51D* transgene. The presence of one, as opposed to two, transgenes in this line facilitated the use of the other transgenes required in these experiments. *Aβ_42_* carrying the E22G (arctic) mutation was used, since a single copy of the wildtype *Aβ_42_* transgene was found not to result in a significant locomotor or survival defects in our experiments. The phenotypes caused by expression of the wild-type Aβ transgene and the arctic Aβ transgene are quantitatively and qualitatively similar, as can be seen from the relative effects of each transgene on locomotor performance and survival (Supplementary Fig. S1). Median survival for the arctic model is 31 days in the Aβ flies compared to 24 days in the 2 × Aβ_42_ model and 66 days in control flies. For the RNAi experiments, *w^1118^, elavGAL4* *> UAS-Aβ_42_arc-51D* flies were used and controls, *w^1118^, elavGAL4/+;51D* (empty insertion site). In these experiments, female virgin *w^1118^; 51D* or *w^1118^; UAS-Aβ_42_arc-51D* flies were crossed to *w^1118^, elavGAL4* *> UAS-RNAi* males. In the PGRP-SC1b experiments (RNAi insertion on chromosome X), female virgin *w^1118^, UAS-PGRP-SC1b RNAi* flies were mated to *w^1118^, elavGAL4* *> UAS-Aβ_42_arc-51D/CyO* or *w^1118^, elavGAL4; 51D/CyO* flies to generate the experimental flies. RNAi and over-expression lines were obtained from the VDRC or Bloomington stock centers. The lines used were Sod3-RNAi (VDRC# 37793, *w_1118_; P{GD4801}v37793*), PGRP-SC1b RNAi (VDRC #51237, *w^1118^, p{GD5490} v51237*), sec31-RNAi (VDRC #35867, *w^1118^; P{GD13867}v35867*), sec31-OE (Bloomington #22308, *y1 w^67^c^23^; P{w[+mC] y[+mDint2] = EPgy2}sec31 [EY19759]*), CG14715 RNAi, (VDRC #104124 *w^1118^; P{KK104150}VIE-260B*; #12828, *w^1118^; P{GD4788}v12828*), CG14715 OE (Bloomington #32608, *w^1118^; P{w[+mC]* *=* *EP}CG14715 [G6908]*). All stocks were backcrossed for at least 6 generations into the *w^1118^* background prior to carrying out the experiments. Flies were raised and maintained on cornmeal medium (87.5 g/l dextrose, 87.5 g/l maize, 19 g/l yeast). Stocks were maintained and experiments were conducted at 25C on a 12:12 hours light/dark cycle at constant humidity.

### Microarray methods

RNA was extracted from 50 fly heads according to standard Trizol (Invitrogen, Paisley, UK) protocols. FlyChip_long_oligonucleotide_003 (FL003) - INDAC (Flychip, University of Cambridge, http://www.flychip.org.uk/) microarray chips were used for the expression analysis (GEO Platform GPL14121). These chips have been used extensively for *Drosophila* expression profiling and include 14,444 transcript-specific oligonucleotides (70mers) and various controls. Samples were labelled and hybridised according to standardised protocols (http://www.flychip.org.uk/protocols/). Four replicates were used for each sample (*w^1118^, elavGAL4* *> UAS-Aβ_42_-51D* (×2 copies) flies and controls *w^1118^, elavGAL4/+; 51D* (×2 copies, empty insertion site) and five time points. Time points used corresponded to days 3,10 and 20 (100% survival in both Aβ and control flies) and days 21, 25 (Aβ flies) or days 56, and 68 (control flies), corresponding to 80% and 20% survival in each case) Samples were matched on arrays by time point. The data discussed in this publication have been deposited in NCBI's Gene Expression Omnibus and are accessible through GEO Series accession number GSE48681 (http://www.ncbi.nlm.nih.gov/geo/query/acc.cgi?acc=GSE48681).

### Analysis of microarray data

The raw data were filtered to remove any probes that were rejected in over 50% of samples and were quantile-normalised across all arrays using Limma^39^. One array (hybridised to a time point 3 sample) was removed from further analysis at this stage. Any missing values were imputed using the impute package (R package version 1.32.0. http://CRAN.R-project.org/package=impute). Differentially expressed genes were identified using two methods. Firstly, Limma was used to fit a linear model to the entire time course and genes identified as significantly differentially expressed were those with an F statistic p-value < 0.05 following FDR correction. Secondly, the maSigPro^40^ package was used to identify genes with significantly (p < 0.05 after FDR correction) different changes in expression over time. For both methods, the samples were matched by % survival. The results from maSigPro and Limma analysis were combined and the expression of each significant gene averaged over all replicates and standardised (to have a mean of 0 and standard deviation of 1). These data, matched by % survival, were then clustered using the R package Mfuzz^13^, which implements fuzzy c-means clustering^12^. Two clustering parameters are required; the fuzzifier m and the number of clusters c. The appropriate value of m was determined using the Mfuzz function “m.estimate”, c was determined by examining the effect of c on the minimum centroid distance^13^,^41^.

maSigPro^40^ was used to identify genes changing expression over time in Aβ and control flies separately (p < 0.05 after FDR correction). Each of these genes was tested for correlation with % survival using a Pearson Product Moment Correlation Coefficient in R.

### Lifespan

Flies were reared at standard density, allowed to mate for 24 h, sorted by sex, and then transferred to experimental vials at a density of ten female flies per vial. Flies were transferred to fresh vials three times a week, and deaths were scored three to five times a week. Lifespan data were subjected to survival analysis (Log-rank tests) using GraphPad Prism 5 Software (GraphPad Software, Inc).

### Locomotor/climbing assays

The locomotor ability of the flies was assessed in a 1 min negative geotaxis assay as previously described^9^. Ten flies were placed in a plastic 25-ml pipette and knocked to the bottom of the pipette. The number reaching the 10 ml line of the pipette (n^top^) and the number remaining at the bottom (below the 2-ml line) (n^bottom^), after 1 min, were measured. The performance (mobility) index was then calculated as (n^top^ − n^bottom^ + n^total^)/2n^total^. Three to four replicates were used per genotype. Climbing in each pipette was assessed three times and the average performance index for each pipette calculated. Assays were carried out in a well-lit room at a temperature of 23–24°C. The mean of the independent biological replicates for each genotype was plotted with the s.e.m.. Two-tailed Student's *t*-tests (*f*-test for equal variance) were used to identify significant differences at specific time points.

### qRT-PCR

Total RNA was extracted from 20 adult heads per genotype using standard Trizol (Invitrogen, Paisley, UK) protocols. RNA was DNase-treated (Fermentas, Thermo Scientific, UK) and cDNA was prepared using oligo-d(T) primers and a Promega Reverse transcription kit (#A3500) according to the manufacturer's protocol (Promega, Southampton, UK). qRT-PCR was performed using a Biorad iQ machine and KAPA (KK4608) SYBR green PCR master mix (Biorad, Hemel Hempstead, UK). Relative quantities of transcripts were determined using the relative ΔΔCt method and normalised to *Act5C*. Two to five independent RNA extractions were used for each genotype. Primer sequences are available upon request.

## Author Contributions

M.E.G., G.F. and D.C.C. designed the project. M.E.G., H.B., B.F., D.M.B. and E.B. carried out experimental work. D.M.B., G.F. and B.F. carried out bioinformatics analysis with the guidance of S.G.O. G.F., M.E.G., D.M.B., S.G.O., S.R., D.C.C. and H.A.B. wrote the paper.

## Supplementary Material

Supplementary Information Supplementary Info

Supplementary Information Table S1

## Figures and Tables

**Table 1:** Differentially expressed genes selected for follow up experiments and summary of results

Gene	Function	Expression Profile	Effect of RNAi in this work
Sod3-RD, Sod3-RE	Extracellular Cu/Zn superoxide dismutase^21^,^22^,^42^	Over-expressed in Aβ flies. Fig. 3a	Improved climbing and survival in Aβarc flies. No effect on survival of control flies, slight increase in climbing performance. Figure 4
*PGRP-SC1b*	Innate immune response^27^	Over-expressed in Aβ flies. Fig. 3e	Improved climbing and survival in Aβarc flies. No effect in control flies. Figure 5
sec31	ER to Golgi transport^29^	Under-expressed in Aβ flies. Fig. 3f	Increased survival of Aβarc flies, no effect on climbing ability. No effect on survival or climbing performance in control flies. Supplementary Figure S6
CG14715	Intracellular transport and protein folding^31^,^30^	Over-expressed in Aβ flies, Fig. 3d	Negative effect on climbing and survival in both control and Aβarc flies. Supplementary Figure S5
