# Supplementary material for: Identification of novel modifiers of Aβ toxicity by transcriptomic analysis in the fruitfly
Source: Sci Rep. 2013 Dec 16;3:3512. doi: 10.1038/srep03512 (PMC3863820; doi:10.1038/srep03512)
Supplement: Supplementary Information — Supplementary Info [file srep03512-s1.pdf]

Identification of novel modifiers of Aβ toxicity by transcriptomic analysis in the fruitfly

G. Favrin, D. M. Bean, E. Bilsland, H. Boyer, B. E. Fischer, S. Russell, D. C. Crowther, H. A. Baylis, S. G. Oliver, M. E. Giannakou

Supplementary Figures

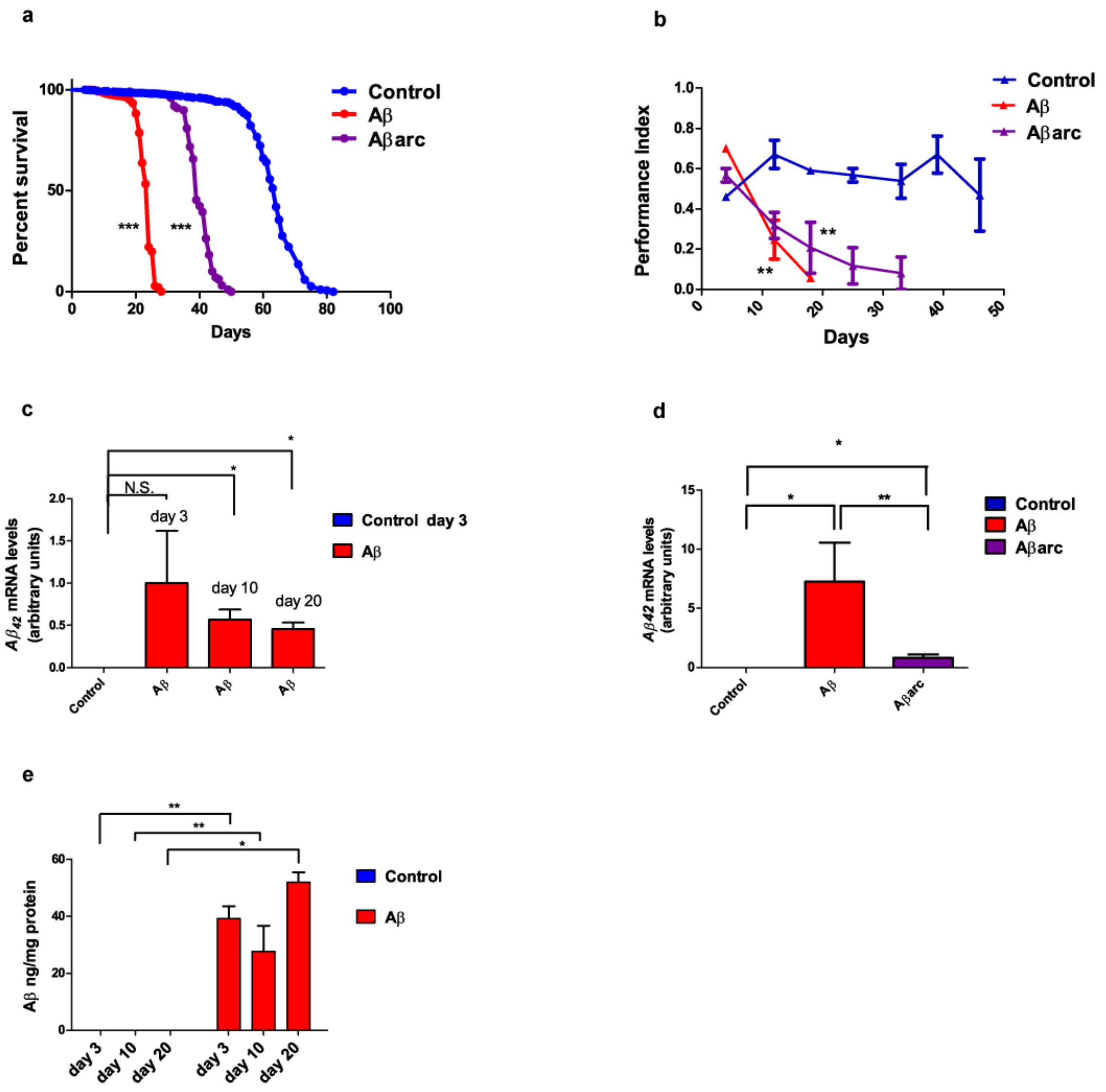

Figure S1. Survival, climbing ability, *Aβ* expression levels and A*β* peptide levels in control and Aβ-expressing flies. (a) Survival curves of Aβ (*elavGAL4> UAS* $A\beta_{42}$ x2, n=*1696,* median=*24, P<0.0001 vs.* Control), Aβarc (*elavGAL4> UAS* $A\beta_{42}$*arc*, n=99*,* m=36*, P<0.0001 vs.* Control) and Control *(elavGAL4/+,* n=*967,* m=*64)* mated female flies at 25°C. Survival curves were compared using Log-rank test. (b) Climbing ability of Aβ, Aβarc and Control mated female flies at various timepoints in adulthood at 25°C (n=30 flies per group). Climbing was assessed using iFly[S1]and comparisons between groups were carried out using linear regression. (c) *Aβ* expression levels in Control and Aβ mated female flies at day 3, 10 and 20, quantified using qRT-PCR relative to *Act5C* expression levels. Levels are represented relative to Aβ_day 3 (arbitrarily set to 1), presented as mean +/- SEM. (d) *Aβ* expression levels in Control, Aβ and Aβarc mated female flies at day 3 of adulthood quantified using qRT-PCR relative to *Act5C* expression levels. Levels are represented relative to Aβarc (arbitrarily set to 1), presented as mean +/- SEM. (e) Total Aβ peptide levels in Aβ and Control flies at day 3, 10 and 20 of adulthood quantified by MSD ELISA (n=3 replicates/group, 10 flies/group). Data presented as mean values +/- SEM. A*β* mRNA and

peptide levels were compared using *t*-test (2-tailed, *f*-test for equal variance). *, P<0.05; **, P<0.01; ***, P<0.0001.

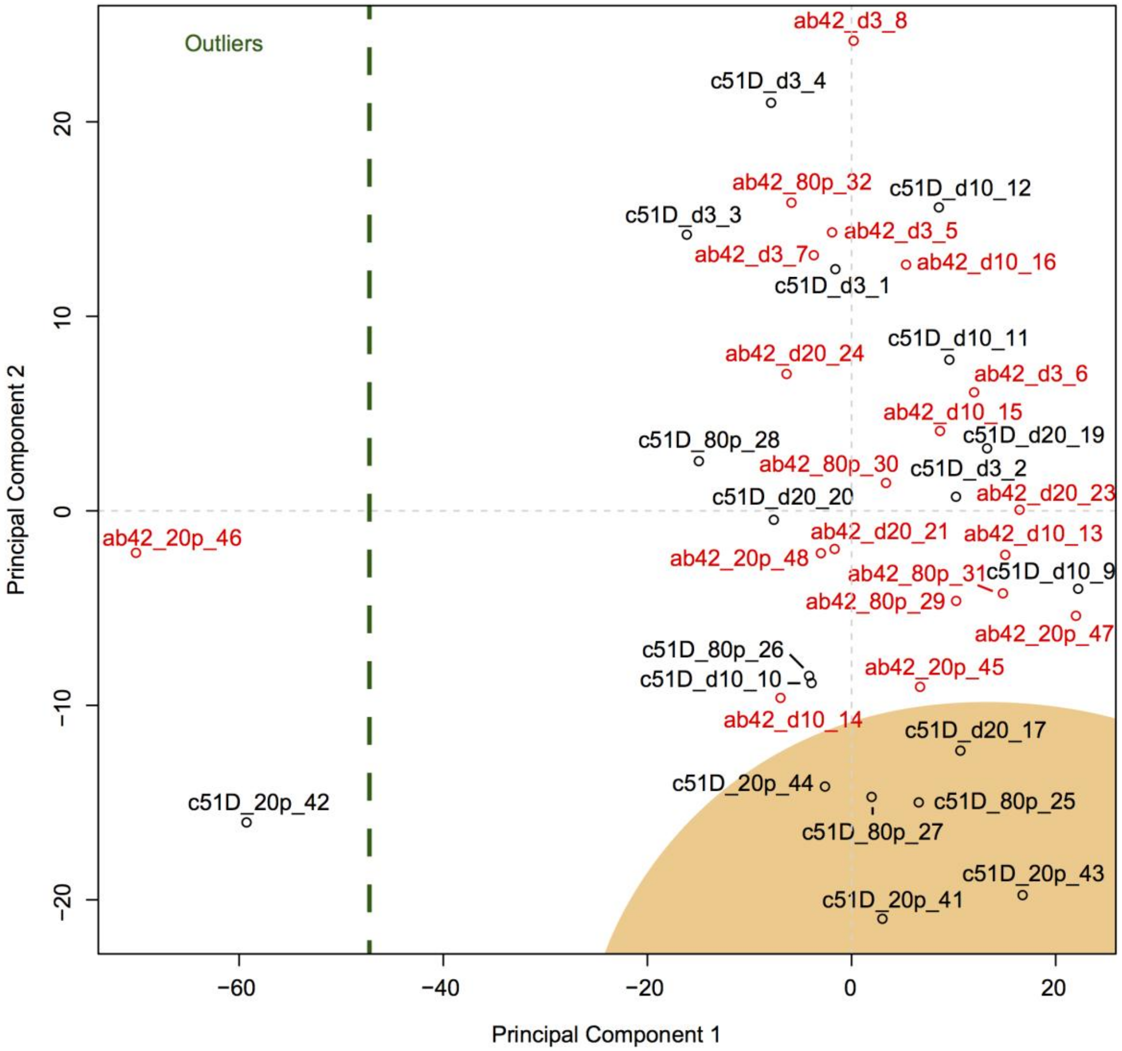

Figure S2. Principal Components Analysis results for all microarray data. On the basis of this result, one array (samples ab42_20p_46 and c51D_20p_42) was excluded from further analysis (beyond the dashed green line). Aβ flies and young controls (days 3, 10, 20) are separated from aged controls (80% and 20% survival; orange region) along component 2. Samples from Aβ flies in red, samples from control flies in black.

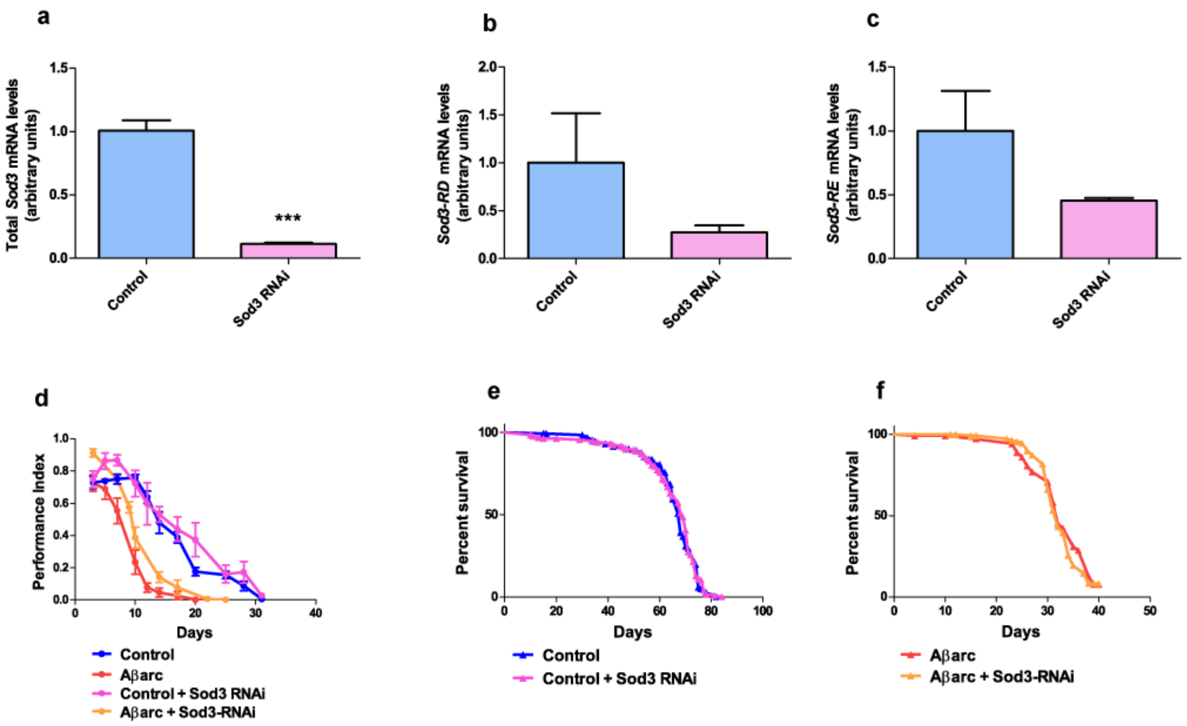

Figure S3. Effects of Sod3-RNAi on *Sod3* expression, locomotion and survival. (a-c) Levels of *Sod3* transcripts in head RNA from flies expressing Sod3-RNAi ubiquitously (with *tubGAL4*) *vs.* control flies at day 3 of adulthood measured by qRT-PCR and plotted relative to *Act5C* mRNA levels, in arbitrary units, represented relative to levels in control (set to 1). *Aβ* mRNA levels were compared using *t*-test (2-tailed, *f*-test for equal variance) and mean values +/- SEM presented. (a) Total *Sod3* mRNA levels. (b) *Sod3*-RD mRNA levels. (c). *Sod3*-RE mRNA levels. (d) Climbing performance of Control (*elavGAL4/+*), Aβarc (*elavGAL4> UAS $A\beta_{42}$arc*), Control+ Sod3 RNAi and Aβarc+ Sod3 RNAi mated females at different timepoints at 24°C. n=3 (3 replicates, 10 flies/replicate). Performance indices (see Methods) between Control and Control+ Sod3 RNAi and Aβarc and Aβarc+ Sod3 RNAi were compared at each time-point using two-tailed Student's *t*- test (*f*-test for equal variance). (e) Survival curves of Control (*elavGAL4/+,* n=124; median survival =68) and Control+ Sod3 RNAi (n=127; m=67, P= 0.9181 *vs.* Control) mated females at 25°C. (f) Survival curves of Aβarc (*elavGAL4> UAS $A\beta_{42}$arc*, n=143, median=32, P<0.0001 *vs.* Control) and Aβarc+ Sod3 RNAi (n=125, median survival =32, P=0.2142 *vs.* Aβarc) mated females at 25°C. Comparison of survival curves was carried out using the Log-rank test. P values: ***, P<0.0001.

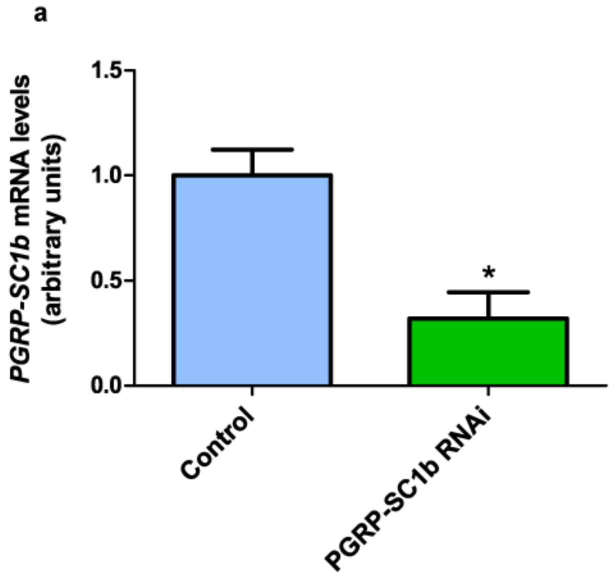

Figure S4. Effect of PGRP-SC1b RNAi on PGRP-SC1b expression. (e) PGRP-SC1b RNA levels in head RNA from flies expressing PGRP-SC1b RNAi ubiquitously (with *tubGAL4*) *vs.* control flies at day 3 of adulthood measured by measured by qRT-PCR and plotted relative to *Act5C* mRNA levels, in arbitrary units, represented relative to mRNA levels in control flies (set to 1). A*β* mRNA levels were compared using *t*-test (2-tailed, *f*-test for equal variance) and mean values +/- SEM presented. P values: *, P<0.05; **, P<0.01; ***, P<0.0001.

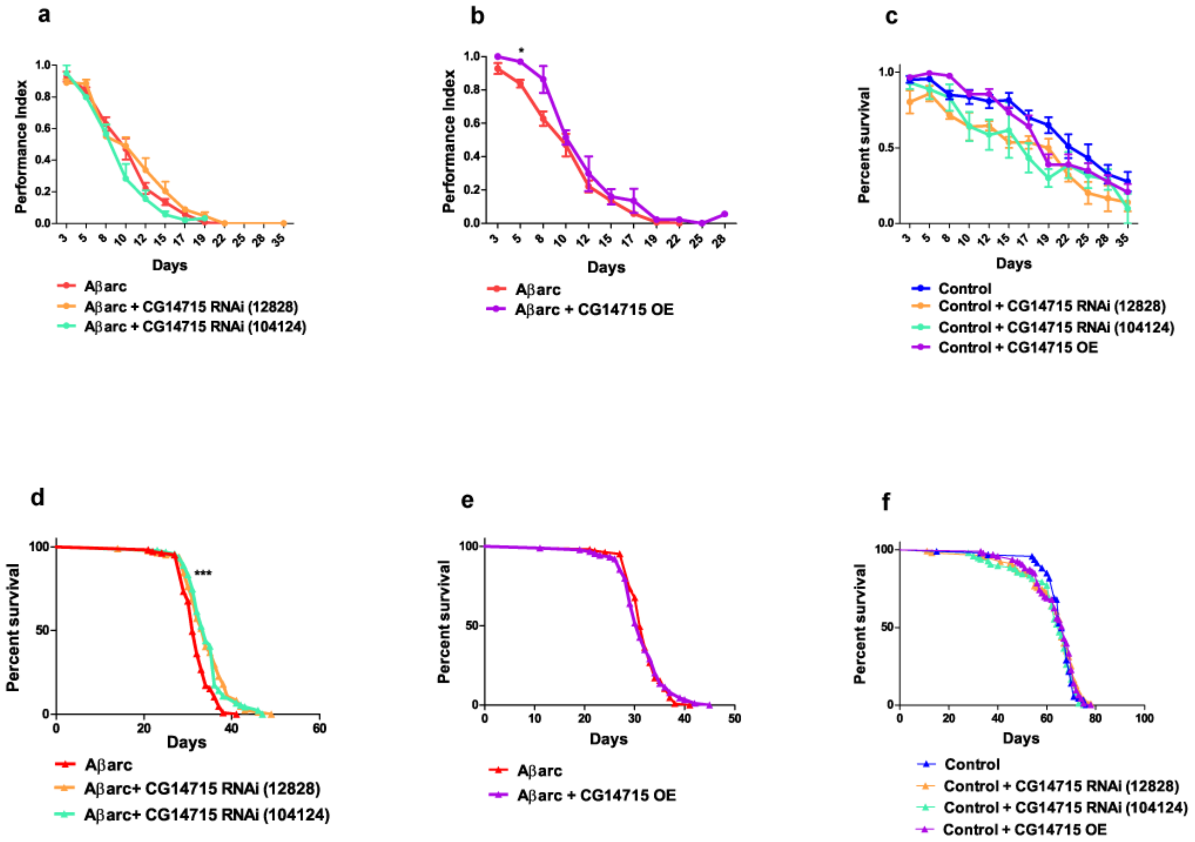

Figure S5: Effects of CG14715-RNAi and over-expression (OE) on locomotion and survival. (a-c). Climbing performance of mated female flies at different timepoints at 24°C (3 replicates, 10 flies/replicate). Performance indices between the two groups (experimental and control) were compared at each time-point using two-tailed Student's *t*- test (*f*-test for equal variance). (a) Climbing performance of Aβarc (*elavGAL4>UAS* $A\beta_{42}$*arc)* and Aβarc+ CG14715-RNAi (RNAi, #104124 or #12828) mated females. (b) Climbing performance of Aβarc and Aβarc+ CG14715-OE mated females. (c) Climbing performance of Control (*elavGAL4/+)*, CG14715-RNAi (RNAi, #104124 or #12828) and CG14715-OE mated females. (d-f) Survival curves. Comparison of survival curves was carried out using the Log-rank test. (d) Survival curves of Aβarc (n=105, median=31), Aβarc+ CG14715-RNAi-104124 (n=91, m=34, P<0.0001 *vs.* Aβarc) and Aβarc+ CG14715-RNAi-12828 (n=84, m=34, P<0.0001 (*vs.* Control)) mated females at 25°C. (e) Survival curves of Aβarc (n=105, median=31) and Aβarc + CG14715-OE (n=89, m=31, P=0.9431 *vs.* Aβarc) mated females at 25°C. (f) Survival curves of Control (n=93, median=66), CG14715-RNAi-104124 (n=96, m=65, P=0.4746 *vs.* Control), CG14715-RNAi-12828 (n=102, m=66, P=0.4954 *vs.* Control) and CG14715-OE (n=93, m=67, P=0.5980 *vs.* Control) mated females at 25°C. P values: *, P<0.05; **, P<0.01; ***, P<0.0001.

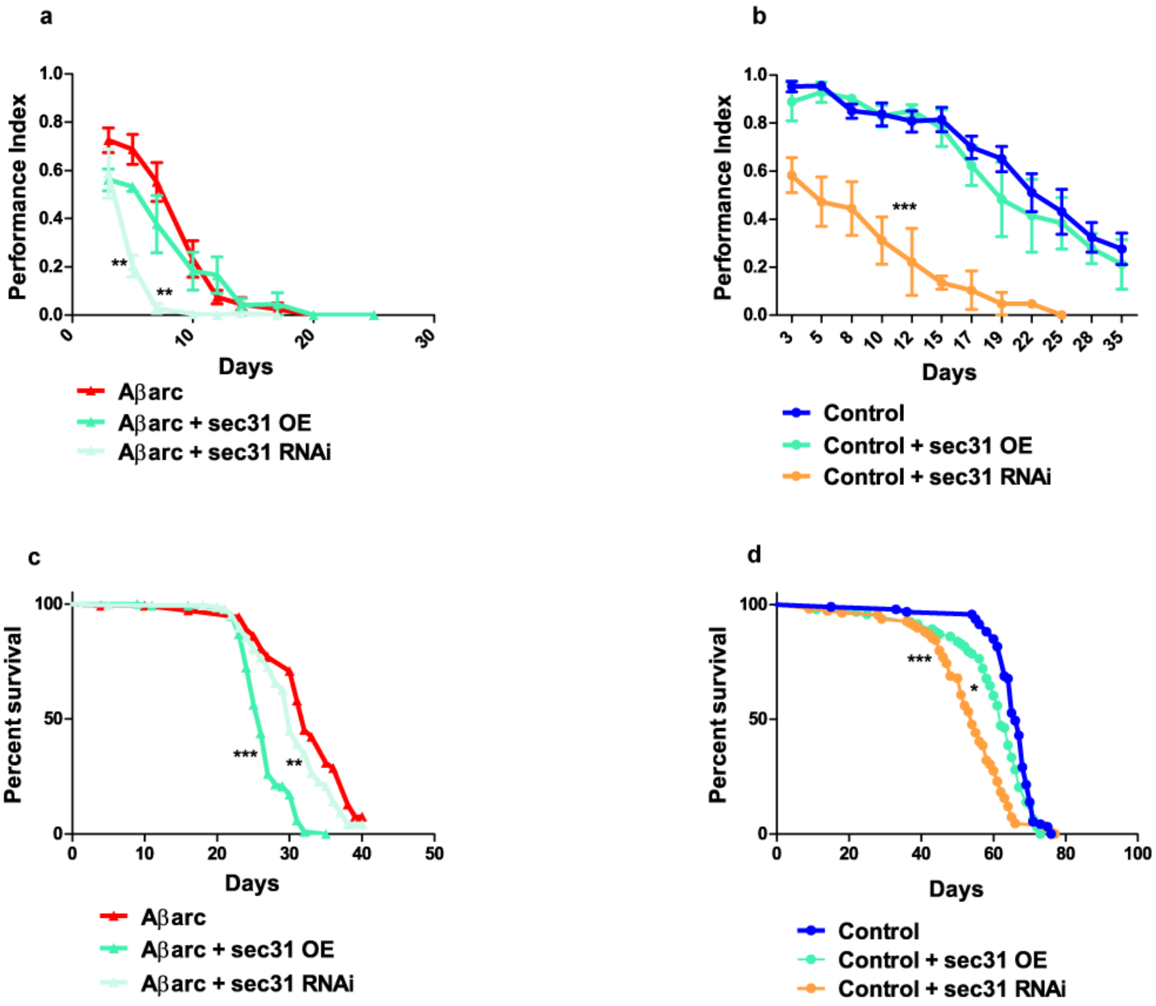

Figure S6. Effects of *sec31* RNAi and over-expression (OE) on locomotion and survival. (a, b) Climbing performance of mated female flies at different timepoints at 24°C (3 replicates, 10 flies/replicate). Performance indices between the two groups were compared at each time-point using two tailed Student's *t*- test (*f*-test for equal variance). (a) Climbing performance of Aβarc (*elavGAL4>UAS* $A\beta_{42}$*arc)*, Aβarc+ sec31 RNAi and Aβarc+ sec31 OE mated females. (b) Climbing performance of Control (*elavGAL4/+)*, Control+ sec31 RNAi and sec31 OE mated females. (c, d) Survival curves. Comparison of survival curves was carried out using the Log-rank test. (c) Survival curves of Aβarc (n=146, median=32), Aβarc+ sec31 RNAi (n=146, m=30, P=0.0005 *vs.* Aβarc) and Aβarc+ sec31 OE (n=137, m=26, P<0.0001 *vs.* Aβarc) mated females at 25°C. (d) Survival curves of Control (n=93, median=66), sec31-RNAi (n=109, m=54, P<0.0001 *vs.* Control) and sec31 OE (n=93, m=62, P=0.028 *vs.* Control) mated females at 25°C. P values: *, P<0.05; **, P<0.01; ***, P<0.0001.

Supplementary methods

Locomotor assays

Locomotor assays in Fig S1 were carried out using iFly[S1].

MSD-ELISA. Protein samples containing total Aβ42 were prepared as described in [50]. Quantification of Aβ42 in the protein samples was carried out using an ELISA assay with reagents from Meso Scale Discovery (Rockville, MD, USA) and read using an MSD ELISA plate reader. Briefly, a 96 well Avidin or Strepavidin plate was pre-coated with 3 % w/v MSD-Blocker A in PBS overnight at 4°C. Between each of the next steps the plate was washed with 0.05 % v/v PBS-Tween. The plate was incubated for 1 hr at RT with primary antibody, 6E10-Biotinylated (Cambridge Biosciences, UK), followed by 1.5 hr at RT incubation of Standard Curve, water, and samples. Finally 1 hr at RT incubation with secondary antibody, 21F12 SULFO- TAG labelled 93 (Elan Pharmaceuticals, USA) was carried out. The plate was read using an MSD plate reader and MSD Reading buffer and later analysed with MSD software. Protein concentrations were quantified using DC protein assay (Bio-Rad Laboratories Ltd., Hemel Hempstead, UK) according to the manufacturers' recommendations.

Supplementary References

S1. Jahn, T. R. *et al.* Detection of early locomotor abnormalities in a Drosophila model of Alzheimer's disease. *J Neurosci Methods* **197**, 186-189, doi:10.1016/j.jneumeth.2011.01.026 (2011).
